# Estimating δ^15^N fractionation and adjusting the lipid correction equation using Southern African freshwater fishes

**DOI:** 10.1371/journal.pone.0178047

**Published:** 2017-05-24

**Authors:** Geraldine C. Taylor, Jaclyn M. Hill, Michelle C. Jackson, Richard A. Peel, Olaf L. F. Weyl

**Affiliations:** 1 South African Institute for Aquatic Biodiversity (SAIAB), Grahamstown, South Africa; 2 Department of Ichthyology and Fisheries Science, Rhodes University, Grahamstown, South Africa; 3 Department of Zoology & Entomology, Rhodes University, Grahamstown, South Africa; 4 Department of Life Sciences, Imperial College London, Ascot, Berkshire, United Kingdom; 5 NNF/EU Community Conservation Fisheries in KAZA Project, Katima Mulilo, Namibia; University of Hyogo, JAPAN

## Abstract

Stable isotope analysis is an important tool for characterising food web structure; however, interpretation of isotope data can often be flawed. For instance, lipid normalisation and trophic fractionation values are often assumed to be constant, but can vary considerably between ecosystems, species and tissues. Here, previously determined lipid normalisation equations and trophic fractionation values were re-evaluated using freshwater fish species from three rivers in the Upper Zambezian floodplain ecoregion in southern Africa. The parameters commonly used in lipid normalisation equations were not correct for the 18 model species (new *D* and *I* parameters were estimated as *D* = 4.46‰ [95% CI: 2.62, 4.85] and constant *I* = 0 [95% CI: 0, 0.17]). We suggest that future isotopic analyses on freshwater fishes use our new values if the species under consideration do not have a high lipid content in their white muscle tissue. Nitrogen fractionation values varied between species and river basin; however, the average value closely matched that calculated in previous studies on other species (δ^15^N fractionation factor of 3.37 ± 1.30 ‰). Here we have highlighted the need to treat stable isotope data correctly in food web studies to avoid misinterpretation of the data.

## Introduction

Stable isotope analysis is a popular tool for analysing the trophic ecology of individuals, populations and communities [1]. Stable carbon (δ^13^C) and nitrogen (δ^15^N) isotopic composition reflects the assimilated food intake of an organism over a given time and can therefore be used to describe food web structure [1–6]. Despite recent advances in the field, there are certain assumptions which need to be met when applying isotope tools where information is still lacking. For instance, defining a fractionation factor, the changes in δ^13^C and δ^15^N between prey and predator, is essential for tracing energy flows and sources, determining trophic position and calculating food chain length [7]. Fractionation factors vary across a number of scales, from ecosystem (marine and freshwater), to taxon (fish and invertebrates), feeding strategy (herbivores and carnivores), species, and even tissue types within species.

Varying significantly with the photosynthetic pathway of primary producers, δ^13^C values are conserved throughout trophic transfers. On average δ^13^C values display a 0–1.5‰ enrichment between consumer and food source, thus preserving information on primary producers at the base of the food web [2,4,5,8]. Comparatively, δ^15^N values increase predictably in a step wise fashion (enrichment of 3‰) with trophic transfers as a result of the retention of heavier isotopes and the excretion of lighter isotopes [1,3,5,9]. This allows inferences to be made about the trophic position of consumers [6], as well as adding information on food sources [10]. However, variation in fractionation has been widely documented. For example, Vander Zanden and Rasmussen [7] found that carnivorous fishes demonstrated a significantly higher δ^15^N fractionation of 3.2‰ compared to the 2.5‰ of herbivorous fishes, while Post [6] found that herbivorous fishes and detritivorous fishes exhibited higher δ^13^C fractionation than carnivorous fishes (0.50 vs. 0.05‰). Hussey et al. [11] developed a scaled δ^15^N fractionation framework from a meta-analysis of experimentally derived fish fractionation studies, concluding that δ^15^N fractionation decreases with increased δ^15^N. Gorokhova and Hansson [12] found δ^15^N fractionation factors of 3.6‰ and 2.7‰ for two different species of mysid shrimps. Sweeting et al. [13] found that muscle tissue had higher δ^15^N fractionation than heart and liver tissue in European sea bass. These differences in fractionation arise as a result of unequal assimilation of dietary components, changing of dietary components by animal tissues, and the differential allocation of nutrients in the diet to different tissues [14]. Although average fractionation factors of 3.4‰ for δ^15^N [6,9] and 1‰ to 1.5‰ for δ^13^C [2,13] are used as standard estimates, ecosystem or species-specific fractionation factors should be estimated whenever possible [8,12,14,15], especially when using mixing models to infer diet [16].

Fish store lipids in multiple organs, including skeletal muscle, and the levels of lipid within fish tissue can vary widely with and among species and in space and time [17–19]. The lipid content of fish muscle tissue affects δ^13^C values because lipids are ^13^C depleted relative to proteins and carbohydrates, complicating the isotopic relationship between a consumer and its dietary sources [20,21]. As the presence of lipids affects δ^13^C and not δ^15^N, there is a well-documented relationship between the amount of lipid a sample contains and it’s C:N ratio [22]. Accounting for lipids in animal tissue can be addressed via chemical extraction or mathematical correction. Extraction however, is expensive, time consuming, and more importantly affects nitrogen isotope values, whereas mathematical normalisation is cheap and is usually sufficient to account for lipid bias in fish tissues [23], however standard validated methods are lacking [24].

Using freshwater fishes from the Upper Zambezian floodplain rivers, the aim of this study was to firstly re-evaluate the parameters *D* and *I* of the McConnaughey and McRoy [22] lipid normalisation equation described for marine organisms. This equation uses the proportions of C and N in the sample (C:N) to i) estimate the lipid content (*L*) of the sample: $L=\frac{93}{1+{\left(0.246\;\times \;\left(C:N\right)-0.775\right)}^{-1}}$, and ii) correct the δ^13^C value to produce the lipid normalised value δ13C’=δ13C+D×(I+3.91+287L). Parameter *D* refers to the isotopic difference between protein and lipid, while *I* is a constant which defines the C:N ratio before which no lipid is extractable. Secondly, this study aimed to estimate a δ^15^N fractionation factor for these freshwater fishes.

## Methods

### Sampling

**Fig 1:**
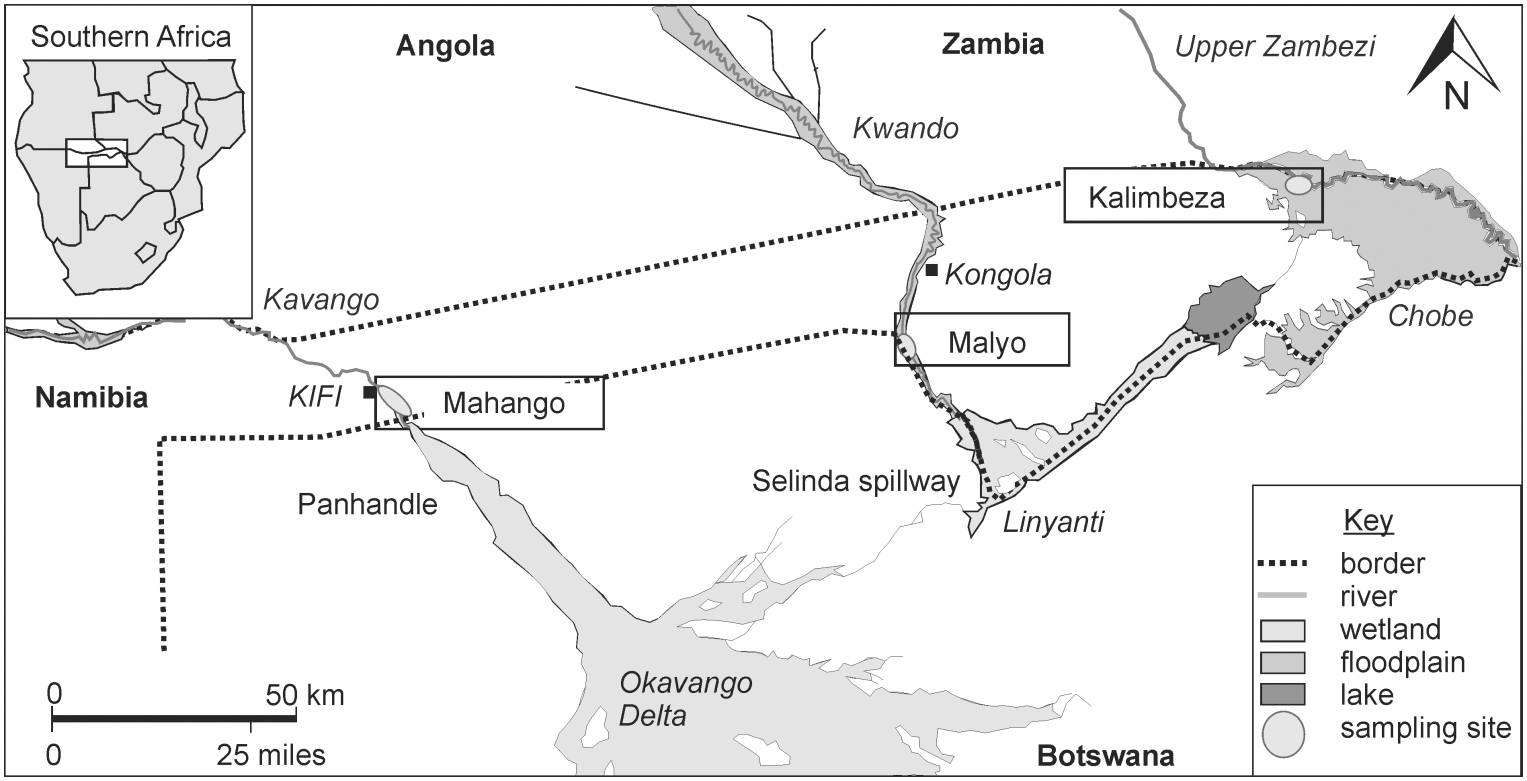
A map of the sample sites on the Upper Zambia, Kavango and Kwando rivers in Namibia.

Samples were collected for stable isotope analyses from the Upper Zambezi (Kalimbeza Channel), Kavango (Mahango National Park) and Kwando (about 5 km either side of Malyo) rivers, Namibia (Fig 1). Samples included: fish white muscle tissue, whole bodies of insects and shrimps, muscle tissue of molluscs and crabs, fresh plant leaves, detritus, and particulate organic matter (POM). Fishes were collected by angling, D-netting, gillnets, fyke nets and long lines. D-nets were of 3 mm mesh size, experimental multifilament gillnets were comprised of randomised 10 m panels of 12, 16, 22, 28, 35, 45, 57, 73, 93, 118 and 150 mm stretched diamond mesh approximately 2.5 m deep. Double ended fyke nets had 1.2 m D-openings and 25 mm mesh size, and baited longlines were 20 m and contained 20 × 9/0 circle hooks on 80 lb monofilament snoods of 1 m in length. Most fish from the gill nets were dead on retrieval, and any live fish were sacrificed by concussion followed by destruction of the brain. Ethical approval was granted by the SAIAB Animal Ethics Committee (Reference # 2013_07). Insects and shrimps were collected using the D-net, molluscs and detritus were collected by hand and using a dredge, crabs were collected by hand and using rod and line at night, while plants were collected by hand. Particulate organic matter (POM) was collected by filtering river water through 0.45 μm pre-combusted (6 hours at 500°C) Advantec glass fibre filters.

### Isotopic analysis

All samples, aside from detritus and POM, were rinsed in water and dried at 50°C for 24–48 hours until constant weight. All plant and detrital material was acid-washed in 1% HCl to remove carbonates which may be enriched in ^13^C, before oven drying [2,25,26]. All samples were then individually crushed to a homogenous powder with a pestle and mortar and weighed into tin capsules. The Zambezi River and Kwando River samples were analysed using a Europa Scientific INTEGRA isotope ratio mass spectrometer at IsoEnvironmental cc, SAIAB, Grahamstown. Analytical precision for δ^13^C and δ^15^N were <0.15‰ and <0.18‰ respectively and isotopic values were normalised to internal standards (beet sugar, ammonium sulphate, casein) and calibrated against International Atomic Energy reference materials (IAEA-CH-3 and IAEA-CH-6 for δ^13^C, IAEA-N1 and IAEA-N2 for δ^15^N). Kavango River samples were analysed on a Flash EA 1112 Series coupled to a Delta V Plus stable light isotope ratio mass spectrometer via a ConFlo IV system (all equipment supplied by Thermo Fischer, Bremen, Germany), housed at the Stable Isotope Laboratory, Mammal Research Institute, University of Pretoria. Analytical precision was <0.18‰ for δ^13^C and <0.12‰ for δ^15^N and isotopic values were normalised to internal standards (Merck Gel and casein) and calibrated against International Atomic Energy reference materials (IAEA-CH-6 for δ^13^C and IAEA-N2 for δ^15^N). All isotopic values are expressed relative to Vienna PeeDee Belemnite and atmospheric nitrogen for δ^13^C and δ^15^N respectively, in standard delta (δ) notation: $\;\delta X\left(‰\right)=\left(\frac{R_{sample}}{R_{standard}}-1\right)\times 1000$, where *X* = ^13^C or ^15^N, *R* = ^13^C/^12^C or ^15^N/^14^N.

### Lipid correction

Three individuals from 18 of the most common fish species from the Kavango River, Namibia were collected in August 2014 (Table 1). Lipids were removed from fish white muscle tissue samples after homogenisation (drying and grinding) using a modified version of the Bligh and Dyer [27] chemical extraction methods. Samples were rinsed in a 2:1 ratio of chloroform: methanol and vortexed for 1 minute to ensure homogenisation, and then centrifuged for 10 min at 10000 rpm. The supernatant was then discarded and the entire procedure was repeated until the supernatant was completely clear and colourless following centrifugation (minimum 3 rinses). Samples were then dried at 60°C for 24 hours to remove remaining solvent, reground and weighed into tin capsules for subsequent isotopic analysis.

**Table 1:** The difference between lipid treated and untreated freshwater fish white muscle tissue samples. Untreated and lipid treated δ^13^C and C:N values, the difference between them (± SD), and the number of individuals subject to lipid extraction (sample size *N*), for 18 fish species from the Kavango River.

Species	*N*	Untreated		Lipid treated		δ^13^C_treated_ - δ^13^C_untreated_
		δ^13^C (‰)	C:N	δ^13^C (‰)	C:N	
*Micropanchax johnstonii*	3	-21.32 ± 0.60	4.25 ± 0.17	-20.23 ± 0.72	3.93 ± 0.03	1.10 ± 0.14
*Brycinus lateralis*	3	-22.39 ± 2.20	4.23 ± 0.38	-21.81 ± 2.03	3.96 ± 0.01	0.58 ± 0.17
*Enteromius poechii*	3	-19.12 ± 0.40	3.93 ± 0.21	-18.55 ± 0.63	3.94 ± 0.02	0.58 ± 0.25
*Clarias gariepinus*	3	-22.47 ± 0.88	3.69 ± 0.09	-21.91 ± 0.73	3.94 ± 0.01	0.56 ± 0.16
*Clarias ngamensis*	3	-21.82 ± 0.54	4.05 ± 0.14	-20.77 ± 0.65	3.97 ± 0.01	1.05 ± 0.12
*Marcusenius altisambesi*	3	-23.32 ± 2.75	4.12 ± 0.55	-22.70 ± 3.29	3.99 ± 0.00	0.62 ± 0.55
*Hepsetus cuvieri*	3	-22.68 ± 0.66	3.84 ± 0.05	-22.01 ± 0.71	3.91 ± 0.04	0.67 ± 0.28
*Hydrocynus vittatus*	3	-22.64 ± 0.40	3.81 ± 0.12	-21.89 ± 0.46	3.89 ± 0.04	0.75 ± 0.06
*Micralestes acutidens*	3	-20.92 ± 0.58	3.99 ± 0.10	-20.13 ± 0.59	3.94 ± 0.04	0.79 ± 0.19
*Oreochromis andersonii*	3	-29.77 ± 0.69	4.41 ± 0.68	-27.97 ± 0.44	3.98 ± 0.03	1.80 ± 0.98
*Oreochromis macrochir*	3	-28.24 ± 0.42	4.44 ± 0.40	-26.14 ± 0.71	3.97 ± 0.03	2.11 ± 0.61
*Pharyngochromis acuticeps*	3	-22.15 ± 1.25	3.93 ± 0.15	-21.46 ± 1.28	3.91 ± 0.01	0.69 ± 0.16
*Petrocephalus okavangensis*	3	-24.75 ± 0.65	4.18 ± 0.15	-23.65 ± 0.50	3.99 ± 0.07	1.10 ± 0.32
*Serranochromis altus*	3	-24.11 ± 2.45	4.01 ± 0.21	-23.32 ± 2.66	3.97 ± 0.02	0.79 ± 0.54
*Serranochromis macrocephalus*	3	-23.63 ± 0.49	3.97 ± 0.13	-22.83 ± 0.53	3.94 ± 0.00	0.80 ± 0.04
*Schilbe intermedius*	3	-21.38 ± 1.38	3.84 ± 0.26	-20.83 ± 1.38	3.93 ± 0.04	0.55 ± 0.04
*Synodontis nigromaculatus*	2	-24.16 ± 3.09	3.84 ± 0.01	-23.34 ± 3.05	3.96 ± 0.02	0.82 ± 0.04
*Coptodon rendalli*	3	-19.66 ± 1.11	4.39 ± 0.26	-18.44 ± 1.26	3.99 ± 0.01	1.22 ± 0.25

The lipid extracted data were used to amend the McConnaughey and McRoy [22] normalisation equation parameter *D* (isotopic difference between protein and lipid) and constant *I*. This normalisation equation uses the proportions of C and N in the sample (C:N) to i) estimate the lipid content (*L*) of the sample: $L=\frac{93}{1+{\left(0.246\;\times \;\left(C:N\right)-0.775\right)}^{-1}}$, and ii) correct the δ^13^C value to produce the lipid normalised value δ13C’=δ13C+D×(I+3.91+287L).

Values for *D* and *I* of 6‰ and -0.207 respectively (22)] are commonly used, however Kiljunen et al. [24] re-estimated them for a suite of brackish water fishes as 7.018‰ (*D)* and 0.048 (*I)*.

For this study the McConnaughey and McRoy [22] model was evaluated, and new *D* and *I* values were estimated from the Kavango River data. For each individual fish white muscle tissue sample, differences between chemically lipid-extracted δ^13^C_treated_ and untreated δ^13^C values (observed), and between normalised δ^13^C’ and untreated δ^13^C values (predicted), were plotted against the untreated C:N ratios. *D* and *I* were estimated by minimising the binomial negative log-likelihood function using: $-LL=Nln\left(\hat{\sigma }\right)$, where $\hat{\sigma }$ is the maximum likelihood estimate of the model standard deviation described as: $\hat{\sigma }=\sqrt{\frac{\sum_{i}{\left(L_{i}-{\hat{L}}_{i}\right)}^{2}}{N}}$, and ${\hat{L}}_{i}$ is the predicted maximum δ^13^C’ at C:N ratio, *L*_*i*_ is the observed δ^13^C lipid treated at C:N ratio and *N* is the total number of lipid treated samples. The variability of the parameters was estimated using the conditioned parametric bootstrap resampling technique described by Efron [28] (N = 1000 iterations).

### Estimation of δ^15^N fractionation values (Δδ^15^N)

The Δδ^15^N values for this study were calculated using identified stomach contents data from *Brycinus lateralis*, *Clarias gariepinus*, *Clarias ngamensis*, *Schilbe intermedius* and *Serranochromis macrocephalus* from the Upper Zambezi, Kavango and Kwando rivers. These species were chosen as they were abundant, found in a variety of habitats and representative of various foraging modes. Fish were collected using gillnets and baited longlines from the Zambezi in October 2013 and July 2014, from the Kavango in June and August 2014, and from the Kwando in August 2013, January—April and July 2014. Live fish were pithed, and all fish were measured to the nearest mm fork length or total length and dissected. For *C*. *gariepinus*, *C*. *ngamensis*, *S*. *intermedius* and *S*. *macrocephalus*, stomach contents were identified, counted and weighed to the nearest 0.1 g after blotting dry on tissue. Bait from longlines was excluded from the stomach contents. *Brycinus lateralis* were preserved in formalin, and in the laboratory stomach contents were identified under a dissecting microscope, counted and weighed to the nearest 0.01 g after blotting dry. Fish remains were identified to species level, while insect remains were identified to family, and non-identifiable remains were excluded from these analyses.

Δδ^15^N fractionation factors were calculated according to the method of Sherwood and Rose [29], using: $\Delta \delta^{15}N=\delta^{15}N-\;\left(\sum_{i=1}^{n}P_{i}\times \delta^{15}N_{i}\right)$, where *δ*^*15*^*N* is the average *δ*^*15*^*N* value of the consumer from a specific population, *P*_*i*_ is the mass proportion of the *i*th prey item in the diet of the consumer from that population, and *δ*^*15*^*N*_*i*_ is the average *δ*^*15*^*N* value of the *i*th prey item from the population sampled. Diet proportions (*P* values) for the stomach contents identified to family for fish and order for invertebrates were determined as: $P_{i}=\;\frac{W_{i}}{W_{tot}}$, where *W*_*i*_ is the total weight (g) of prey *i* consumed by all of the individuals of the consumer from a given population, and *W*_*tot*_ is the total weight (g) of all prey consumed by the consumer from the same population.

## Results

### Lipid correction

The δ^13^C and C:N values of the 18 fish species white muscle tissue samples analysed from the Kavango River varied both within and between species (Table 1). After lipid extraction, δ^13^C values were higher for all species, and for those species where C:N > 4 before treatment, extractions resulted in a decrease in both C:N ratios and variability. However, pre lipid extraction C:N values did not vary as much as those sampled in the literature [24], suggesting the lipid content of the species sampled was not high or particularly variable.

**Fig 2:**
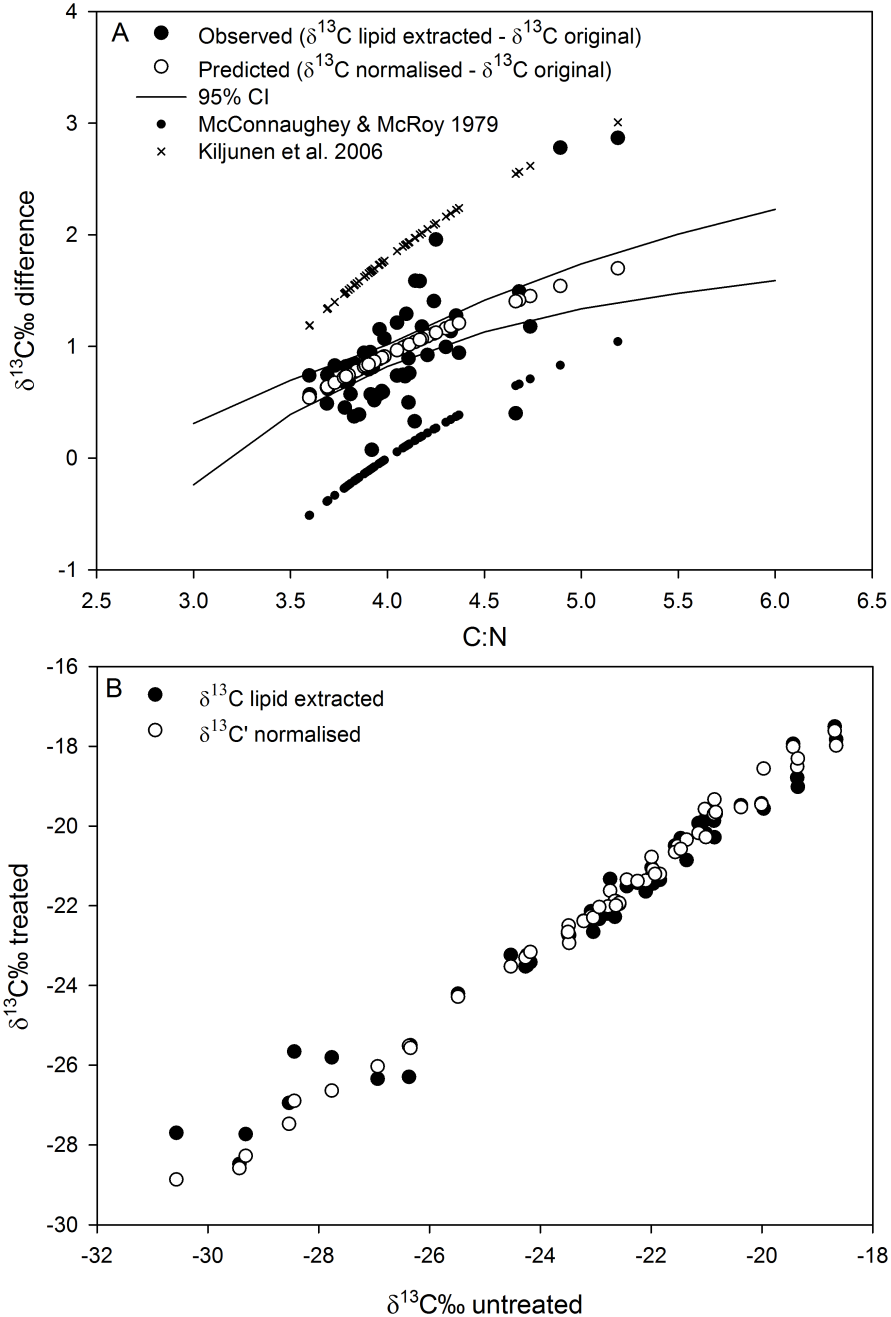
Re-evaluating the parameter *D* and constant *I* for the lipid normalisation equation. A: the difference in δ^13^C between lipid-extracted and δ^13^C untreated values, and δ^13^C’normalised and δ^13^C untreated values in relation to the C:N ratio of white muscle tissue of freshwater fishes. This is compared with the lipid normalisation equation estimated by McConnaughey and McRoy [22] and re-evaluated by Kiljunen et al. [24]. B: The δ^13^C lipid extracted and δ^13^C’ normalised values in relation to the δ^13^C untreated values. This illustrates the accuracy of the amended normalisation equation in calculating δ^13^C’ values which coincide with the δ^13^C lipid extracted samples.

The predicted relationship (difference between δ^13^C’normalised and δ^13^C untreated values) for the differences between δ^13^C lipid-extracted and δ^13^C untreated values, did not fit the McConnaughey and McRoy [22] or the Kiljunen et al. [24] formulae (Fig 2A). New *D* and *I* parameters were estimated (parameter *D* = 4.46‰, 95% CI: 2.62, 4.85; and constant *I* = 0, 95% CI: 0, 0.17), which fit the observed data (*R*^2^ = 0.41, *d*.*f*. = 51, *p* < 0.001), so that δ^13^C’normalised and δ^13^C lipid treated values coincided (Fig 2B). The amended relationship intersected the x-axis at 3, suggesting that fish muscle contained zero extractable lipid at a C:N ratio of 3.

### Estimation of Δδ^15^N

An average Δδ^15^N factor of 3.37‰ (±1.30) was calculated (Table 2) from species from each river system which had N > 20 stomachs containing identifiable stomach contents (Table 3). If the fractionation factors are broken down by river, the highest and lowest fractionation factors in the Zambezi River were seen for *S*. *intermedius* (3.19‰) and *C*. *gariepinus* (2.23‰), *C*. *gariepinus* (5.17‰) and *S*. *intermedius* (1.62‰) in the Kavango River and *C*. *ngamensis* (5.29‰) and *B*. *lateralis* (3.3‰) in the Kwando River, respectively (Table 2).

**Table 2:** The fractionation factors (Δδ^15^N) estimated using stomach contents analysis of predatory freshwater fish species from the Upper Zambezi, Kavango and Kwando rivers. N_stomachs_ are the number of stomachs which contained identified prey items used for this analysis, and δ^15^N the isotopic values used for the analyses. The average and standard deviation of Δδ^15^N has been calculated from the Δδ^15^N per species by river as detailed in the table.

River	Species	N_stomachs_	δ^15^N‰	Δδ^15^N‰
Zambezi	*Serranochromis macrocephalus*	21	7.74	2.57
Zambezi	*Schilbe intermedius*	55	7.17	3.19
Zambezi	*Clarias gariepinus*	27	8.62	2.23
Kavango	*Schilbe intermedius*	114	6.43	1.62
Kavango	*Clarias ngamensis*	28	7.68	2.72
Kavango	*Clarias gariepinus*	55	9.13	5.17
Kwando	*Clarias ngamensis*	32	8.71	5.29
Kwando	*Clarias gariepinus*	26	9.03	4.44
Kwando	*Brycinus lateralis*	41	5.77	3.13
			Average	3.37 ± 1.30

**Table 3:** The mass proportions (×100) of identified stomach contents for a number of fish species from the Zambezi (Zam), Kavango (Kav) and Kwando (Kwa) rivers, and their *δ*^*15*^*N*_*i*_ values.

	*Clarias gariepinus*			*Clarias ngamensis*		*Serranochromis macrocephalus*	*Schilbe intermedius*		*Brycinus lateralis*	*δ*^*15*^*N*_*i*_‰		
	Zam	Kav	Kwa	Kav	Kwa	Zam	Zam	Kav	Kwa	Zam	Kav	Kwa
**Arthropods**												
Odonata	0.10	-	0.25	1.14	3.76	-	3.71	0.43	-	3.11	3.91	4.38
Araneae	-	-	-	1.90	-	-	-	-	-	-	6.69	-
Coleoptera	-	-	-	6.17	-	-	-	0.10	0.23	-	4.34	2.73
Ephemeroptera	-	-	-	0.33	2.02	-	2.97	0.01	89.66	2.09	0.64	2.79
Diptera	-	-	-	0.38	0.09	-	-	0.01	0.25	-	1.35	3.48
Hemiptera	-	-	-	0.09	4.42	-	-	-	-	-	4.88	4.25
Oligochaeta	-	-	-	-	0.47	-	-	-	-	-	-	0.73
Trichoptera	-	-	0.25	-	13.59	-	-	-	3.91	-	-	2.88
**Fishes**												
Alestidae	0.39	-	1.25	-	-	6.50	3.22	1.03	-	7.09	6.26	6.08
Cyprinidae	0.25	-	0.50	-	-	27.64	32.66	-	-	6.42	-	5.71
Cichlidae	8.02	2.26	40.63	18.42	5.83	26.29	9.40	2.56	-	6.61	5.70	6.67
Distichodontidae	-	-	-	0.09	-	3.25	0.74	-	-	6.42	6.32	-
Clariidae	51.58	-	-	1.38	-	-	-	0.56	-	7.48	8.56	-
Mormyridae	-	6.80	0.25	-	-	16.26	11.38	78.50	-	6.06	5.62	6.33
Siluridae	-	12.06	-	-	-	-	-	1.71	-	-	6.43	-
Synodontidae	28.38	37.13	-	28.72	-	-	-	-	-	6.88	6.44	-
**Other**												
Shrimp	-	-	-	1.54	1.60	-	1.46	0.00	-	5.22	3.88	3.86
Crab	-	4.35	35.62	23.45	40.45	-	-	0.00	-	-	6.04	4.89
Mollusca	-	-	-	0.26	9.41	-	-	-	-	-	3.01	2.49
Detritus	-	14.56	0.13	3.96	11.29	-	1.76	0.82	-	2.36	0.17	-0.37

## Discussion

The results presented here demonstrate that, for lipid normalisation, the standard use of the McConnaughey and McRoy [22] formulae may be inaccurate without the re-estimation of the parameter *D* and constant *I*. In the present study the McConnaughey and McRoy [22] formula underestimated the difference between δ^13^C lipid corrected and δ^13^C untreated values, while the Kiljunen et al. [24] formula overestimated the difference between δ^13^C lipid corrected and δ^13^C untreated values. These differences may be attributed to the ecosystems sampled, since freshwater fishes were sampled in this study, while marine organisms were sampled by McConnaughey and McRoy [22], and brackish water fishes were sampled by Kiljunen et al. [24]. The causal machanisms of these differences may stem from variations in lipid metabolism in marine, brackishwater and freshwater fishes, a concept that should be explored in future research. We suggest that future isotopic analyses on freshwater fishes use the parameter *D* = 4.46‰ and constant *I* = 0 estimated here, if the species do not have a high lipid content in their white muscle tissue indicated by C:N ratio of 5 or lower. Future research should reassess the McConnaughey and McRoy [22] lipid normalisation formulae using lipid extracted and non-lipid extracted samples of freshwater fishes with a large range of white muscle lipid contents.

The mechanisms behind the isotopic enrichment between consumer and food source, or fractionation, are not well understood [14], yet the estimation of fractionation factors is important when using stable isotopes to construct food webs and evaluate trophic dynamics [8]. The use of experimental studies to estimate fractionation factors is time consuming and involves the collection of newly hatched or juvenile fishes, and their long term survival and successful feeding in captivity [8]. Since it is accepted that the analysis of both stomach contents and stable isotopic ratios are complementary [30–32]: the former having a high taxonomic resolution, while only providing a snapshot of dietary information at one point in time [33]; and the latter having low taxonomic resolution [34] while estimating the assimilated dietary inputs over a longer period of time [1–6]; it makes sense to use the knowledge acquired using stomach contents analysis to estimate fractionation factors for stable isotope analysis. The downfalls of this approach include the high sample sizes required for stomach contents analysis to accurately measure fish diets, especially when using predators where many stomachs are empty [32,35,36].

In addition, since fractionation factors are necessary for estimating at what trophic level food sources lie, one must assume that all food sources lie at a similar trophic level, when calculating fractionation factors. For example, *Clarias gariepinus* from the Zambezi River feed on a range of organisms including Ondonates, a family of invertebrates which are likely to occupy a lower trophic level than Clariid fishes, on which *C*. *gariepinus* feed cannibalistically. Despite these shortcomings, the average Δδ^15^N of 3.37‰ calculated in this study, is similar to both fractionation factors estimated using experimental studies [9,15] and those collated from the literature [6–8,37]. A literature review by Sweeting et al. [8] of 56 experimental studies published between January 1977 and November 2005, as well as his experimental study on European sea bass, estimated a similar average Δδ^15^N of 3.15‰ for fish muscle tissue. Vander Zanden and Rasmussen [7], working in 20 lakes in Ontario and Quebec, found that piscivorous fishes had a similar Δδ^15^N of 3.49‰, while Post [6] found that herbivorous fishes (3.35‰) had a lower Δδ^15^N than carnivorous fishes (3.45‰) using literature on 25 northern temperate lakes. Alternatively, Pinnegar and Polunin [38] found that rainbow trout had a Δδ^15^N of 2.55‰, while McCutchan et al. [15] completed a literature survey and found that the average Δδ^15^N for aquatic organisms was 2.3‰. Thus the variation in fractionation factors indicates that there is a degree of uncertainty around which food web studies described using stable isotope analyses are constructed. Future studies should aim to validate these fractionation factor results using experimental studies [14] in tropical and sub-tropical systems in the southern hemisphere, where literature is lacking.
